# Epidemiology of injuries in male under-19 futsal players competing in the highest national league in Spain

**DOI:** 10.3389/fspor.2026.1830511

**Published:** 2026-06-24

**Authors:** Oscar Villanueva-Guerrero, Bruno Travassos, Mário Lopes, Rafael Albalad-Aiguabella, Hadi Nobari, Elena Mainer-Pardos

**Affiliations:** 1 Health Sciences Faculty, Universidad San Jorge, Villanueva de Gállego/Zaragoza, Spain; 2 Research Center in Sports Science, Health Sciences and Human Development, CIDESD, University of Beira Interior, UBI, Covilhã, Portugal; 3 Portugal Football School, Portuguese Football Federation, Oeiras, Portugal; 4 Institute of Biomedicine and School of Health Sciences, University of Aveiro, Aveiro, Portugal; 5 LFE Research Group, Department of Health and Human Performance, Faculty of Physical Activity and Sport Science (INEF), Universidad Politécnica de Madrid, C/Martín Fierro 7, Madrid, Spain; 6 Department of Exercise Physiology, Faculty of Educational Sciences and Psychology, University of Mohaghegh Ardabili, Ardabil, Iran

**Keywords:** injury incidence, injury risk, sports injuries, team sport, young players

## Abstract

**Introduction:**

This study analyzed injury epidemiology and incidence in male youth futsal players competing in the top national category over a regular season. The aim was to quantify injury incidence, prevalence, and burden, and to describe injury distribution according to exposure (training vs. match), body region, injury type, severity, and playing position.

**Methods:**

A prospective cohort study was conducted during the 2023–2024 season in the Spanish Youth Division of Honour, the highest national Under-19 futsal league in Spain. The sample comprised 164 male players from 13 teams who were followed prospectively over a nine-month period. Injury data were recorded throughout the season, whereas training and match exposure were estimated based on the standard schedule of the participating teams. Playing position, dominant leg, injured body region, injury type, severity, and time-loss duration were also recorded. Injury incidence, prevalence, and burden were subsequently calculated.

**Results:**

A total of 111 injuries were registered, affecting 79 players (48% of the cohort), resulting in an overall incidence of 4.96 injuries per 1,000 hours of exposure. Match incidence (53.84/1,000 h) was substantially higher than training incidence (1.94/1,000 h). Most injuries involved the lower limbs (80.1%), particularly the ankle (23.4%) and knee (20.7%). Muscle/tendon (36.9%) and ligament (36.0%) injuries were the most frequent, and wingers sustained the highest number of injuries (*n* = 51). Median time loss was 16 days, while mean time loss reached approximately 36 days, and injury burden reached 176.5 days per 1,000 hours.

**Conclusion:**

These findings indicated a considerable injury risk in this population, especially during matches, and supported the need for targeted prevention strategies focused on strength development, neuromuscular control, and load management.

## 1 Introduction

Futsal is a high-intensity team sport in which injuries frequently occur with different levels of severity, leading to temporary or prolonged unavailability of players for training and competition (1, 2). Given its confined indoor environment and constant high-intensity play, futsal is recognized as one of the highest risk team sports for injury (3, 4). Its reduced 40 × 20 m playing area and the intensely intermittent nature of match play may further contribute to this elevated risk (1). At higher competitive levels, the combination of repeated high-intensity movements performed during both training and match play, together with congested training and competition schedules and frequent exposure to contact situations, may place players at an even greater risk of injury. Before implementing injury prevention programmes into everyday futsal training routines, it is therefore essential to establish the magnitude of the problem in terms of the incidence, severity, and characteristics of injuries (3). As in other team sports, injuries in futsal arise from the interaction of multiple factors, including modifiable factors such as physical fitness and non-modifiable factors such as age and genetics (5, 6).

Players in developmental stages represent a particularly sensitive developmental stage, characterized by ongoing growth, biological maturation, and transient alterations in neuromuscular control and coordination, which may increase susceptibility to both traumatic and overuse injuries (7). In this context, epidemiological studies in youth athletes are particularly relevant, as they help identify the magnitude, characteristics, and distribution of injuries during a key period of athletic development (7, 8). Beyond the physical consequences, injuries can also generate significant economic costs for players, clubs, and federations, highlighting the need for further research on the epidemiology of injuries in specific sports and across different competitive levels (9).

In futsal, injury epidemiology has been extensively investigated in adult and elite players, consistently reporting high injury incidence rates and identifying the lower extremities as the most frequently affected anatomical regions, particularly the groin, knee, and hamstrings (10–12). For example, studies conducted in professional futsal have reported overall injury incidences of approximately 4.5 injuries per 1,000 hours of exposure (13), with hamstring injuries being among the most common, reaching incidence rates of up to 1.80 injuries per 1,000 hours of exposure (11). Furthermore, pooled evidence from a systematic review and meta-analysis in elite male futsal players reported an overall injury incidence of 6.8 injuries/1,000 h and a match injury incidence of 44.9 injuries/1,000 h, highlighting the substantial injury risk associated with the sport (12). Despite the growing body of evidence in adult populations, there is still a lack of research focused on competitive youth futsal players. Preliminary data suggest that injury incidence may also be considerable in younger cohorts. For instance, an under-21 futsal tournament reported an incidence of 292 injuries per 1,000 match-hours (14). However, comprehensive, season-long epidemiological studies in youth players competing at high levels are still scarce, highlighting the need for further research in this specific population.

Despite the growing popularity of futsal among youth athletes worldwide, injury research in developmental stages remains scarce (15). Understanding the epidemiology of injuries in this population is essential to develop effective preventive strategies and promote safe athletic development (16). Given the expanding evidence on the effectiveness of prevention programs in youth sports, it is necessary to tailor interventions to the specific demands of futsal (3). Moreover, little is known about how injury patterns in youth futsal might differ between training and match play, an aspect that could inform context-specific prevention measures. Therefore, the main objective of this study was to analyze the epidemiology, incidence, and characteristics of injuries in youth futsal players over a regular season. A secondary aim was to examine injury patterns according to the context of exposure (training vs. matches) and playing position, in order to provide a more detailed and applied epidemiological profile of injuries in this population.

## 2 Materials and methods

### 2.1 Study design and subjects

A prospective, descriptive cohort study was conducted over the 2023–2024 season with male U19 futsal players competing in the Spanish Youth Division of Honour, the top national U19 futsal league in Spain, classified as tier 3 according to the participant classification framework (17). All 14 teams invited from the Spanish Youth Division of Honour agreed to participate. However, one team was excluded due to the absence of injury monitoring throughout the season. The final sample included 164 players from 13 teams. The distribution of players by playing position was as follows: goalkeepers (*n* = 26), defenders (*n* = 34), wingers (*n* = 75), and pivots (*n* = 29). The mean age of the participants was 17.12 ± 0.94 years. Throughout the regular competitive season, training exposure was estimated based on the standard schedule followed by the participating teams, which typically consisted of three weekly training sessions of approximately 90 min each, plus one official match on weekends, within a regular league of 30 matches. Training exposure was estimated at team level according to this standard schedule and the number of players included in each team. Only exposure during the regular season (preseason not included) was included in the analysis, and friendly matches were not included. The inclusion and exclusion criteria were as follows: (1) all players actively competing in the league were included; (2) players who were injured at the beginning of the season were included from the date of full recovery; and (3) for players who still had an ongoing injury at the end of the season, time-loss was estimated using the average duration observed for that specific injury type.

This study was approved by the Ethics Committee for Clinical Research of the Autonomous Community of Aragón (C.P.–C.I. PI24/137). All participants were informed about the objectives of the study, and written informed consent was obtained. In the case of minors, consent was provided by their legal guardians. All procedures were conducted in accordance with the principles outlined in the Declaration of Helsinki.

### 2.2 Exposure and injury registration

Injuries were recorded daily by each team's coaching staff, including the date of injury onset and the date of return to full participation, while the lead investigator (OV-G) conducted weekly follow-ups to ensure data accuracy and consistency. Before data collection, the coaching staff were instructed on how to record injuries using the predefined IOC consensus categories. For more severe injuries, the diagnosis and time-loss classification were defined by federation medical staff. The injury definition used in this study followed the International Olympic Committee Consensus Statement: “An injury is tissue damage or other derangement of normal physical function due to participation in sports, resulting from rapid or repetitive kinetic energy transfer” (8). All injuries were categorized according to the classifications outlined in the 2020 International Olympic Committee (IOC) Consensus Statement (18). The following variables were recorded for each injury: playing position, affected body region, injury type, time-loss duration, time of the season, and the athlete's dominant leg. Injury severity was determined using time loss, defined as the number of days the athlete is unavailable for training or competition, from the injury onset until the athlete was able to return to full participation without restrictions (8). Total player exposure was calculated from estimated training exposure and official match exposure. Given the rolling substitutions in futsal and the absence of individual minute-by-minute match participation data, match exposure was estimated at team level using the official match duration and the number of on-court players per team. The total exposure time recorded was 22,360 hours, consisting of 21,060 hours of training and 1,300 hours of competition.

### 2.3 Statistical analysis

**Figure 1:**
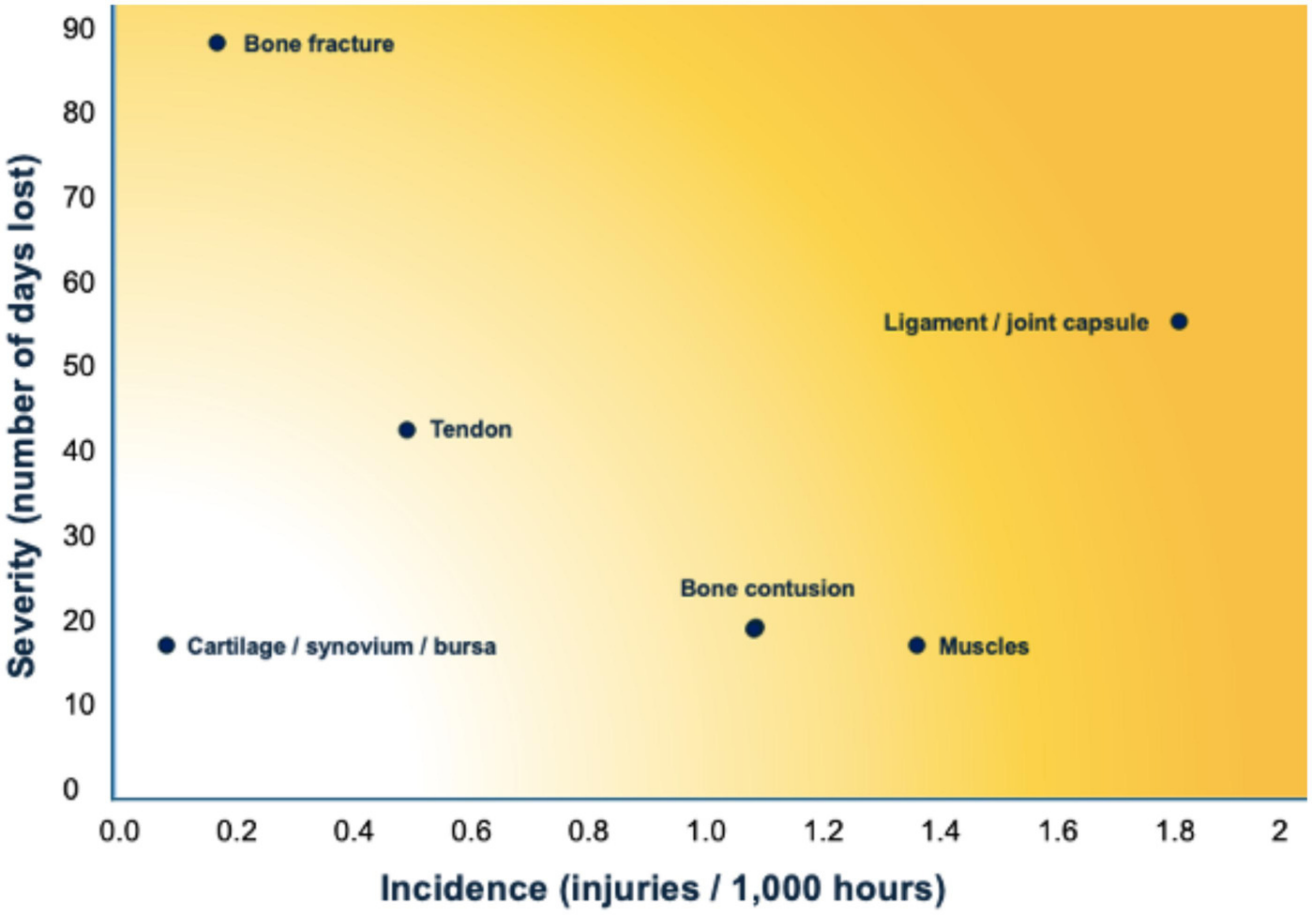
Quantitative injury risk matrix by injury type. Association between injury incidence and average time- loss in days.

**Figure 2:**
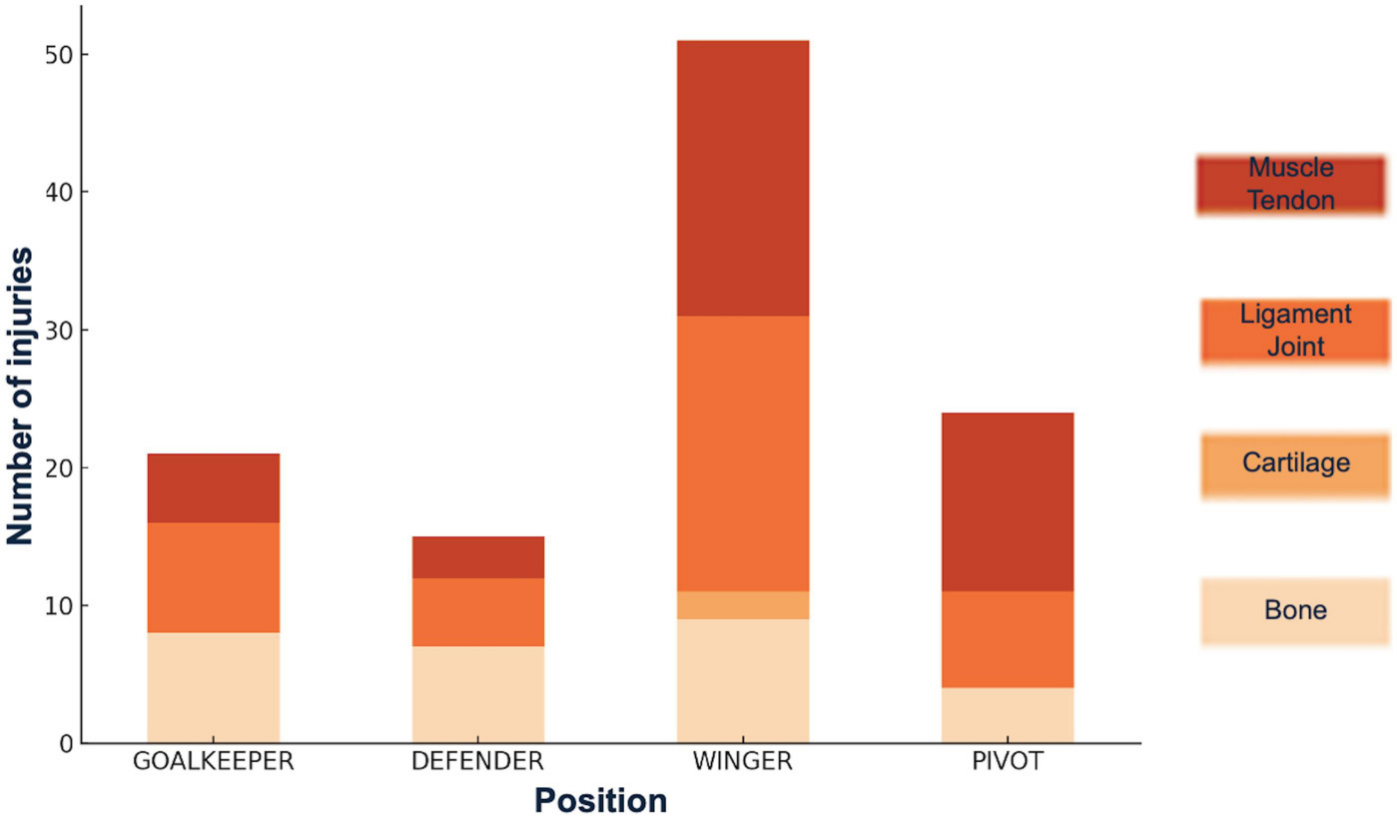
Distribution of injuries by playing position and grouped injury category.

**Figure 3:**
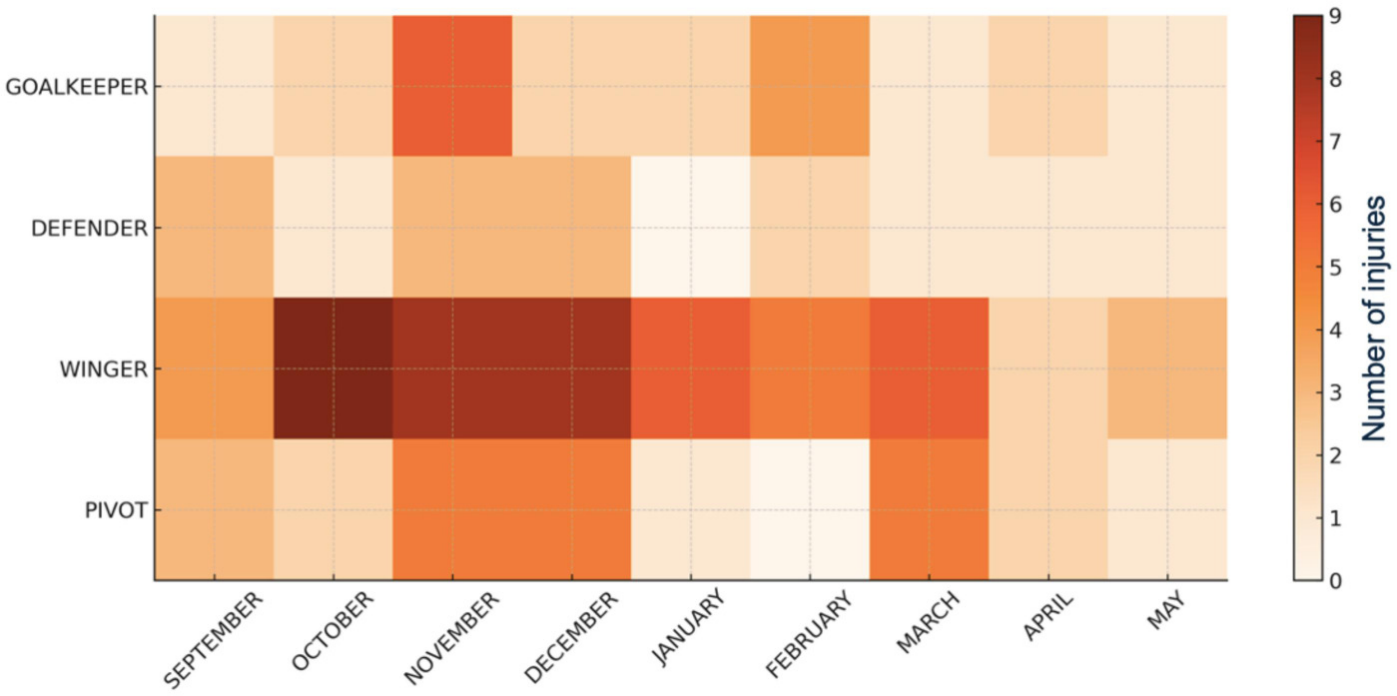
Monthly injury distribution by playing position throughout the 2023–2024 season.

Descriptive statistics were used for data analysis. Categorical variables were presented as absolute frequencies and percentages. Time-loss data were primarily reported as median and interquartile range (IQR). Injury prevalence was calculated as the number of injured players divided by the total number of participants. Injury incidence was reported as the number of injuries per 1,000 hours of exposure, distinguishing between training and competition. Injury burden was calculated as the total number of time-loss days per 1,000 hours of exposure (8). The 95% confidence intervals (CI) for injury incidence were estimated using a Poisson distribution. In contrast, 95% CI for injury burden were estimated using a bootstrap resampling procedure based on 1,000 iterations, following recent methodological recommendations (19). In each iteration, the observed time-loss values for each injury category were resampled with replacement, and burden was recalculated as the total number of time-loss days divided by total exposure hours and multiplied by 1,000. The 2.5th and 97.5th percentiles of the resulting bootstrap distribution were used to define the 95% CI. As individual exposure time was not available for each injury record, injury burden values were derived using total exposure time. When reporting data by injury severity, the following time-loss categories were used: 0 days (slight), 1–7 days (mild), 8–28 days (moderate), and >28 days (severe) (2). Additionally, a quantitative injury risk matrix by injury type was developed to visually represent the relationship between injury incidence and severity across the most common injury types (Figure 1). A stacked bar chart was also created to compare the frequency and distribution of grouped injury categories across playing positions, providing insight into the positional injury burden (Figure 2). A heatmap was also generated to display the monthly distribution of injuries by playing position throughout the season (Figure 3).

## 3 Results

### 3.1 Incidence and characterization of injuries

Over the season, 79 of the 164 players sustained at least one injury (prevalence 48%). In total, 111 injuries were recorded, corresponding to an overall incidence of 4.96 injuries per 1,000 hours of exposure (95% CI: 4.06–5.90). Notably, match injury incidence (53.84/1,000 h) (95% CI: 41.98–68.03) was approximately 28-fold higher than training injury incidence (1.94/1,000 h) (95% CI: 1.40–2.64). In total, 71 injuries occurred during matches and 40 during training. The median time-loss per injury was 16.00 days (IQR: 9.00–30.00), while the mean time-loss was 36 days. When analyzing injuries by playing position, wingers sustained the highest number of injuries (*n* = 51), followed by pivots (*n* = 24), goalkeepers (*n* = 21), and defenders (*n* = 15).

### 3.2 Injury location and type

Injury location and type are presented in Table 1. Most injuries (80.1%) affected the lower extremity. The most frequently injured regions were the ankle (23.4%) and the knee (20.7%), followed by the thigh and foot (both 13.5%). Regarding injury type, the most common were muscle and tendon injuries (36.9%), followed by ligament/joint capsule injuries (36.0%), and bone contusions (21.6%).

**Table 1:** Classification of injuries by type and location .

	N (%)	Incidence Injuries/1,000 h (95% CI)	Median Time Loss (IQR)	Total Days Lost	Burden (95% CI)
INJURY LOCATION					
Head and neck	**1** (**0.9)**	**0.04** (**0.00–0.12)**	**9.00 (N/A)**	**9**	**0.40 (N/A)**
Upper limb	**16** (**14.4)**	**0.72** (**0.36–1.07)**	**16.50** (**12.75–32.00)**	**412**	**18.43** (**11.85–25.94)**
Shoulder	3 (2.7)	0.13 (0.00–0.31)	13.00 (8.00–51.50)	106	4.74 (0.40–12.08)
Elbow	2 (1.8)	0.09 (0.00–0.26)	16.00 (14.50–17.50)	32	1.43 (1.16–1.70)
Wrist	4 (3.6)	0.18 (0.00–0.39)	29.00 (22.25–32.00)	101	4.52 (2.01–6.35)
Hand	7 (6.3)	0.31 (0.07–0.55)	14.00 (12.50–36.50)	173	7.74 (4.07–11.54)
Trunk	**5** (**4.5)**	**0.22** (**0.03–0.42)**	**21.00** (**7.00–25.00)**	**92**	**4.11** (**2.10–6.13)**
Lower limb	**89** (**80.1)**	**3.98** (**3.15–4.81)**	**16.00** (**9.00–30.00)**	**3,435**	**153.62** (**109.92–200.94)**
Hip/groin	6 (5.4)	0.27 (0.05–0.50)	17.50 (13.25–21.00)	107	4.78 (3.80–5.90)
Thigh	15 (13.5)	0.67 (0.28–1.05)	15.00 (7.00–20.50)	270	12.08 (7.33–18.25)
Knee	23 (20.7)	1.03 (0.61–1.45)	33.00 (14.00–146.00)	2,043	91.37 (55.63–131.98)
Lower leg	3 (2.7)	0.13 (0.00–0.29)	14.00 (7.50–88.50)	178	7.96 (0.13–21.87)
Ankle	26 (23.4)	1.16 (0.71–1.61)	14.00 (7.00–19.50)	435	19.45 (13.28–26.79)
Foot	16 (13.5)	0.71 (0.36–1.07)	14.50 (9.25–28.25)	402	17.98 (9.97–28.00)
TYPE OF INJURY					
Muscles/tendon	**41** (**36.9)**	**1.83** (**1.29–2.41)**	**17.00** (**9.00–24.00)**	**967**	**43.24** (**30.23–58.81)**
Muscles	30 (27.0)	1.34 (0.86–1.83)	14.50 (7.25–21.00)	509	22.76 (17.53–30.10)
Tendon	11 (9.9)	0.49 (0.22–0.80)	19.00 (12.00–59.50)	458	20.48 (8.63–33.50)
Bone	**28** (**25.2)**	**1.25** (**0.80–1.74)**	**14.50** (**9.00–38.00)**	**799**	**35.73** (**22.54–52.96)**
Bone fracture	4 (3.6)	0.18 (0.01–0.35)	75.50 (58.75–102.25)	342	15.30 (7.42–24.96)
Bone contusion	24 (21.6)	1.07 (0.67–1.52)	13.00 (8.50–30.00)	457	20.43 (14.31–27.10)
Cartilage/synovium/bursa	**2** (**1.8)**	**0.09** (**0.00–0.22)**	**17.00** (**12.50–21.50)**	**34**	**1.52** (**0.72–2.33)**
Ligament/joint capsule	**40** (**36.0)**	**1.79** (**1.23–2.37)**	**16.00** (**12.00–39.75)**	**2,148**	**96.06** (**56.39–140.88)**
TOTAL	**111** (**100)**	**4.96** (**4.06–5.90)**	**16.00** (**9.00–30.00)**	**3,949**	**176.57** (**133.79–227.90)**

Association between injury incidence and average time-loss in days.

Figure 1 presents the incidence and severity of the most common injury types. Incidence was expressed as the number of injuries per 1,000 hours of exposure, whereas severity was expressed as the total number of time-loss days associated with each injury type. This figure allows a clearer comparison of the frequency and practical impact of each injury type on player availability.

Figure 2 shows the distribution of injuries across playing positions and grouped injury categories. Wingers sustained the highest number of injuries overall, with a particularly high prevalence of muscle/tendon and ligament/joint injuries. Pivots also showed a notable number of muscle/tendon injuries, whereas defenders and goalkeepers presented a more balanced distribution, with a predominance of bone and ligament/joint injuries.

Figure 3 illustrates the monthly distribution of injuries by playing position throughout the 2023–2024 season. The heatmap shows the number of injuries per month for each position (Goalkeeper, Defender, Winger, Pivot), with darker colors indicating a higher injury count. Notably, wingers showed the highest injury burden, particularly during the early phase of the season (September to November).

### 3.3 Severity and burden of injuries

Moderate injuries were the most common (51%), followed by severe (27%) and mild (22%) injuries. The analysis of injury burden revealed a total of 3,949 days of absence due to injury, corresponding to 176.5 time-loss days per 1,000 hours of exposure (95% CI: 133.79–227.90). The injuries associated with the longest recovery periods were bone fractures and ligament/joint capsule injuries.

## 4 Discussion

This study aimed to analyze the epidemiology, incidence, and characteristics of injuries in youth futsal players over a regular season, as well as to examine injury patterns according to playing position and temporal distribution throughout the season. Overall, the findings highlighted a considerable injury burden in this population, with injuries occurring much more frequently during matches than during training sessions, emphasizing the elevated physical and contextual demands of competition. In addition, almost half of the players sustained at least one injury during the season, indicating that injuries represent a relevant issue in male youth futsal. Time-loss data also suggested substantial variability in injury severity, with most injuries resulting in relatively short or moderate periods of absence, whereas a smaller number of severe injuries contributed disproportionately to total time loss. Most injuries affected the lower extremities, particularly the ankle and knee, and muscle, tendon, and ligament injuries were the most frequent types. These findings underline the need for targeted prevention strategies aimed at reducing injury risk and minimizing player unavailability throughout the season.

The injury incidence reported in this study (4.96 injuries per 1,000 hours of exposure) falls within the range previously described in systematic reviews on the epidemiology of injuries in elite futsal (6.8/1,000 h) (12). The match-related incidence observed in our sample was 53.84 injuries/1,000 h, while previous studies in elite futsal have reported values around 44.9/1,000 h, highlighting the substantial impact of competitive intensity on injury risk in youth players (8, 13). Similarly, previous futsal studies have reported that injury rates in matches are an order of magnitude higher than in training sessions (12). This increased incidence during matches may be related to factors such as accumulated fatigue, high-speed directional changes, and unexpected contact situations (20). In fact, the majority of injuries in futsal are caused by contact with another player, with only about one-third resulting from non-contact mechanisms (10). In addition, both internal and external load fluctuate considerably between training and competition, with a greater number of explosive actions and high-intensity efforts during matches, increasing neuromuscular and mechanical stress (21). In youth futsal, the manipulation of game formats according to players’ age can influence internal and external load demands, underscoring the need to carefully manage training loads according to age and maturation status (22). In this context, the implementation of specific prevention programs such as neuromuscular training and exercises targeting stability and postural control has been associated with a significant reduction in injury risk in high-intensity sports like futsal (23, 24). Therefore, it is essential to design strategies tailored to the actual demands of the game in order to reduce injury incidence in youth players and support their physical and performance development (3).

Regarding injury type and location, the results of this study confirm that most injuries affected the lower limbs, with the most commonly injured regions being the ankle, knee, and thigh, which are partially consistent with previous findings in futsal players (1). However, this distribution differs from that typically reported in adult elite populations, where groin and hamstring injuries are often described as the most frequent (10–12). This discrepancy may be explained by the specific characteristics of the study population, as players aged 16–18 years represent a transitional stage between youth and adult performance levels. In this context, ongoing physical development, neuromuscular control, and exposure to different training and match demands may influence injury patterns, which may help to contextualize the distribution of injuries observed in the present cohort. In the present study, the ankle (23.4%), knee (20.7%), and thigh (13.5%) were among the most frequently affected regions; in comparison, previous futsal literature reported values of 23.1%, 12.5%, and 10.3%, respectively. This distribution is also consistent with previous research identifying ankle sprains and knee injuries as common injury patterns in futsal players (12). Studies included in a systematic review on youth football players, also reported similar findings regarding the most common injury types (sprains) and locations (knee and ankle) (25). Moreover, because youth players are still developing, they may be less able to tolerate high training and match loads, so careful load management is especially important in this age group (26, 27). In the present study, ligament, muscle, and tendon injuries represented the most common injury types. This pattern is similar to that reported in professional players from the Portuguese First Division, where ligament-related and muscle-related injuries were also among the most frequent injury categories (13). This similarity suggests that these injury patterns are not exclusive to elite adult athletes, but also significantly affect youth players, highlighting the importance of implementing preventive strategies from early stages of development (28).

When analyzing the monthly incidence of injuries, a considerable increase was observed in November and December. This pattern coincides with that observed in previous studies evaluating the temporal distribution of injury incidence and burden. For example, a study of the Portuguese First Division identified November as the month with the highest number of injuries and December as the month with the highest burden (13). The higher number of injuries recorded during the months of October and November may be explained by the fact that training loads are typically higher during the preseason than during the competitive period (11). As previously noted, this accumulation of physical load and residual fatigue can increase injury risk during the early weeks of competition, particularly if the transition between physical preparation and match-specific demands was not properly managed (29). Managing the transition from preseason to competition is crucial, as abrupt changes in load can elevate injury risk if not properly periodized (30, 31).

When comparing injury distribution by playing position, the results of our study are consistent with the findings of Martínez-Riaza et al. (32), who analyzed injuries over a five-year period in the Spanish national futsal team. Their study reported that wingers sustained the highest number of injuries compared to other positions (50.4%), a pattern also observed in our sample (45.9%). This higher injury rate among wingers may be explained by the greater physical and physiological demands associated with their playing role. Recent studies have shown that wingers are involved in a higher number of high-intensity actions, such as sprints, accelerations, and rapid changes of direction, as well as frequent offensive and defensive transitions (33). While the distribution of injury types was relatively balanced across other positions, wingers showed a clear predominance of muscle/tendon and ligament/joint injuries. These demands, coupled with the fact that the start of the season often coincides with suboptimal levels of physical preparation in many players, may increase neuromuscular fatigue and mechanical stress and could help explain the higher number of injuries in this positional profile during the first months of competition. Early identification of at-risk players and tailored interventions could further contribute to reducing injury incidence (34).

This study has several strengths, including its prospective design, the inclusion of players from multiple clubs, and the analysis of injuries in athletes competing in the highest national U19 futsal league in Spain. Nevertheless, several limitations should be acknowledged. First, injuries were recorded by each team's coaching staff rather than by medical professionals, which could limit diagnostic accuracy despite following IOC guidelines. Although more severe injuries were defined by federation medical staff, not all injuries were systematically verified by medical professionals, which should be considered when interpreting specific anatomical or tissue-level diagnoses. Second, the preseason period was not monitored, so some injuries or load effects prior to the season were not captured. Third, we did not record the mechanism of injuries (e.g., whether injuries were contact or non-contact, or due to overuse), which limits our understanding of how injuries occurred in this cohort. Fourth, the study did not collect data on players’ anthropometric or fitness characteristics, such as body mass index (BMI) or strength levels. This prevented us from analyzing the impact of these factors. These limitations should be considered when interpreting the results of this study.

## 5 Conclusion

This study describes the injury epidemiology of male under-19 futsal players competing in the highest national U19 futsal league in Spain. Overall injury incidence was 4.96 injuries per 1,000 hours, with a much higher incidence in matches than training. Injuries mainly affected the lower limbs (especially ankle and knee), with muscle/tendon and ligament injuries being most frequent. Moderate injuries predominated, indicating a meaningful impact on availability. Injury burden was higher in the early season (notably November–December), and wingers sustained the most injuries. These findings support prioritizing targeted prevention and load management strategies, particularly during high-risk periods and for higher-demand playing roles.

## Data Availability

The raw data supporting the conclusions of this article will be made available by the authors, without undue reservation.
